# Acute kidney disease in patients with COVID-19. A prospective, multicenter, multinational study in Latin America

**DOI:** 10.1590/2175-8239-JBN-2024-0154en

**Published:** 2025-08-18

**Authors:** Raúl Lombardi, Alejandro Ferreiro, Yanissa Venegas, Mariana Pereira, Cristina Carlino, Rolando Claure-Del Granado, Daniela Ponce, Daniel Molina, Agustina Zinoveev

**Affiliations:** 1 Universidad de la República, Facultad de Medicina, Centro de Nefrología, Montevideo, Uruguay.; 2 Universidad Peruana Cayetano Heredia, Hospital Nacional Arzobispo Loayza, Lima, Perú.; 3 Hospital do Servidor Público do Estado de São Paulo, São Paulo, SP, Brazil.; 4 Hospital Provincial de Rosario, Nefrología, Santa Fe, Argentina.; 5 Ministerio de Salud, Centro Único de Donación, Ablación e Implante de Órganos (CUDAIO), Santa Fe, Argentina.; 6 Caja Nacional de Salud, Hospital Obrero No 2, División de Nefrología, Cochabamba, Bolivia.; 7 Universidad Mayor de San Simon, Facultad de Medicina, Instituto de Investigaciones Biomédicas e Investigación Social (IIBISMED), Cochabamba, Bolivia.; 8 Universidade Estadual Paulista, Departamento de Nefrologia, Botucatu, SP, Brazil.; 9 Caja Petrolera de Salud, Santa Cruz, Bolivia.; 10 Centro de Asistencia del Sindicato Médico del Uruguay (CASMU), Institución de Asistencia Médica Privada de Profesionales sin fines de lucro (IAMPP), Departamento de Nefrología, Montevideo, Uruguay.

**Keywords:** Acute Kidney Disease, Acute Kidney Injury, COVID-19, Proteinuria

## Abstract

**Introduction:**

Acute kidney disease (AKD) is defined as functional and/or structural abnormalities of kidneys with health implications and a duration of ≤90 days. This study aimed to evaluate AKD as a more appropriate approach to these conditions for which we used a cohort of COVID-19 patients in whom kidney impairment is expressed by proteinuria and/or loss of function.

**Methods:**

Observational, prospective, longitudinal, multinational cohort study conducted across five Latin American countries. Adult patients with diagnosis of COVID-19 were included. Upon hospital admission, urinalysis or urine strip test was performed. If protein was not detected, a follow-up search was conducted five days later. Patients were classified in four AKD categories: non-kidney disease, proteinuria only, acute kidney injury (AKI) only, and proteinuria and AKI.

**Results:**

Three hundred and sixty patients were included. AKD was present in 273 (75.8%), of whom 142 (52.0%) had only proteinuria, 11 (4.1%) had AKI without proteinuria, and 120 (43.9%) had both proteinuria and AKI. Overall, proteinuria with or without AKI was present in 262 patients (72.8%). AKI with or without proteinuria occurred in 131 patients (36.4%). AKI was mainly severe, non-oliguric, and hospital-acquired. In-hospital mortality increased with the severity of AKD: non-kidney disease 9.5%, proteinuria only 22.8%, AKI only 56.7%, and proteinuria plus AKI 53.0% (p = 0.001).

**Conclusions:**

Our data endorse a comprehensive approach based on the concept of AKD. This integrative approach, encompassing the structural and functional continuum of AKI, AKD, and CKD, enables timely interventions and the implementation of preventive and therapeutic strategies.

## Introduction

The concept of acute kidney disease was introduced for the first time by the KDIGO AKI Workgroup in 2012^1^ with the aim of providing an integrated clinical approach to patients experiencing acute abnormalities in kidney function and structure. The KDIGO Workgroup defines acute kidney disease (AKD) as the occurrence of acute kidney injury (AKI), an eGFR <60 mL/min/1.73 m^2^, a decrease in GFR by >35%, an increase in serum creatinine of >50%, or any kidney damage, within 7 to 90 days following an episode of AKI. AKD is a concept under construction. The initial proposal has been reviewed in 2016 by ADQI Group, proposing a definition and staging and renal recovery criteria and making recommendations for clinical practice and future research^2^. More recently, KDIGO organized a consensus conference with the goal of expanding and harmonizing the existing definitions of AKD. Consequently, the KDIGO guidelines now define acute kidney disease as functional and/or structural abnormalities of the kidneys affecting health and lasting ≤90 days, with AKI being a subset of AKD. Kidney disease is further classified according to its cause, the severity of structural and functional abnormalities, and the duration of these abnormalities^3^. This perspective on acute functional and structural kidney disorders provides a broader understanding of these complex conditions. This innovative perspective aims to bridge the gap between AKI and CKD, thereby identifying patients who do not meet the criteria for AKI and CKD but share the spectrum of baseline disorders. Using to these criteria, it is possible to identify subgroups of patients: AKD without AKI, AKD with AKI, and those with not-known kidney disease (NKD).

Kidney involvement in COVID-19 has been extensively documented and is frequently linked to adverse outcomes^4^. Proteinuria has been reported in 28-84% of cases^5^, while AKI affects approximately 17% of patients, with incidence ranging widely from 0.5% to 80%, as indicated by a systematic review and meta-analysis of 20 cohorts^6^. This clinical profile makes this an appropriate population for the aim of the study. To the best of our knowledge, there are no studies that assess the accuracy of AKD criteria in this setting. Therefore, the purpose of our study was to evaluate AKD criteria as a broader approach to patients with acute/subacute kidney involvement in COVID-19 patients.

## Methods

### Study Protocol

This is an observational, prospective, longitudinal, multinational cohort study conducted from March 8, 2021 to April 30, 2022 across five Latin American countries (Argentine, Bolivia, Brazil, Peru, and Uruguay). All patients who met the inclusion criteria of the study were included. Participants entered the data into a dedicated online platform designed for this study, ensuring the confidentiality of information through data de-identification. Once enrolled in the platform, each patient was randomly assigned a unique identification number, which was used to access their patient-specific information. Upon hospital admission, the presence of proteinuria was assessed either through urinalysis or urine strip tests. Proteinuria was considered positive if the urine protein concentration was ≥ 0.3 g/L in spot urine or ≥ + in urinary dipstick. In cases where no protein was initially detected, a follow-up analysis was conducted five days later. Data recording occurred for all patients, regardless of the presence or absence of proteinuria. Inclusion criteria encompassed adult patients aged ≥18 years, confirmed to have COVID-19 through RT-PCR of nasopharyngeal swabs or antigen tests for SARS-CoV-2 in nasopharyngeal swabs, and requiring hospitalization.

Exclusion criteria consisted of patients with chronic kidney disease (CKD) stage 5, those currently undergoing chronic dialysis, or individuals with functioning transplanted kidneys.

### Definitions

The diagnosis and staging of AKI were determined using the KDIGO 2012 criteria, which define AKI as an increase in serum creatinine (SCr) of ≥0.3 mg/dL within 48 hours or ≥50% above the baseline within the prior 7 days. The most recent outpatient creatinine measurement 365–368 days before admission was considered for baseline. The classification of AKD was based on the KDIGO 2020 definition and classification, which considers abnormalities in kidney function and/or structure with health implications and a duration of ≤3 months^6^. Non-recovery was considered in cases where SCr did not decrease or the patient remained on dialysis, full recovery when SCr decreased to the baseline level or lower, and partial recovery when SCr decreased but did not reach the baseline. We categorized AKI as community-acquired (CA-AKI) if the patient exhibited an elevated SCr within the first 24 hours of admission and hospital-acquired (HA-AKI) when AKI developed during hospital stay.

### Data Collection

Data were obtained from the clinical records of patients. Variables included country and city of residence, demographics, comorbidities, condition at admission, process of care, risk factors and etiology of AKI, in-hospital complications, COVID-19 vaccination status, and discharge condition.

### Statistical Analysis

Quantitative variables are presented as median and interquartile range (IQR) according to their distribution, and the categorical variables as number and proportions. The Kolmogorov-Smirnov test was employed to assess data distribution. For bivariate comparisons between groups, the Chi-square test was applied to categorical variables, and the Mann-Whitney U test or the Kruskal-Wallis test was used for quantitative variables. Odds ratios and 95% confidence intervals (CIs) were calculated. Risk estimation to predict mortality was conducted by calculating the odds ratio (OR) by multivariate stepwise logistic regression model, with the corresponding 95% CI. All statistical tests were two-sided, and significance was determined by a probability of the null hypothesis ≤5%. Data processing and statistical analysis were conducted using IBM SPSS Statistics Base version 22 (NY, USA).

### Ethics Approval and Consent to Participate

The study protocol was approved by the Ethics Committee of the Hospital de Clínicas, School of Medicine, Universidad de la República, Uruguay; the Ethics Committee, Hospital Provincial de Rosario, Sante Fe, Argentina, and Ethics Committee of the Hospital Nacional Arzobispo Loayza, Lima, Peru. Remaining participants were authorized to participate in the study by their Hospital authorities under approval of the aforementioned Ethic Committees. Given the observational nature of the study and the appropriate de-identification of data as mentioned above, informed consent was not deemed mandatory by the reference Ethics Committees, as stated in the approved Study Protocol.

## Results

Three hundred and sixty patients from five countries participated in the study, with the following distribution: Peru (45%), Brazil (22.5%), Bolivia (14.7%), Argentina (11.1%), and Uruguay (6.7%). The median (IQR) age of the cohort was 64 (50–74) years, with a majority of male patients (57.7%). The most prevalent comorbidities included arterial hypertension (49.7%), diabetes (31.8%), and obesity (26.3%). Upon admission, the median (IQR) serum creatinine level was 0.86 (0.68-1.07) mg/dL. Regarding hospitalization, the majority of patients were admitted to the ward (73.8%), with 15.6% in the intensive care unit (ICU) and 10.6% in the emergency room. However, ICU admissions increased to 50.9% during the hospital stay, and 45.3% of patients received invasive mechanical ventilation. Complications were frequent (48.9%), with sepsis being the most common (27.6%). The median hospital length-of-stay was 15 (7–28) days, and the all-cause in-hospital mortality rate was 30.2%. Out of 185 patients (51.4%) for whom vaccination status information was available, 100 (54.1%) had received at least one dose, while 82 of the vaccinated patients received two or three vaccine doses (82%). Table 1 provides a detailed profile of patients according to their vaccination status. Vaccinated patients exhibited fewer comorbidities, less disease severity, particularly in terms of proteinuria and hematuria, a lower rate of ICU admission, and a lower mortality rate. AKI was also less frequent but didn’t reach statistical difference.

**Table 1:** CLINICAL CHARACTERISTICS OF THE ENTIRE POPULATION

Age, years, median (IQR)	64 (50–74)
Male sex %	57.7
Hypertension %	49.7
Diabetes %	31.8
Obesity %	26.3
Chronic heart disease %	11.5
Chronic kidney disease %	4.4
Malignancy %	4.4
Immunodepression %	4.7
Hospital setting % Ward Emergency room ICU	73.8 10.6 15.6
Serum creatinine mg/dL, median (IQR)	0.86 (0.68–1.07)
Lymphocyte count mm^3^, median (IQR)	951 (622–1415)
Platelet count mm^3^, median (IQR)	235 (181–301)
D-dimers ng/mL, median (IQR)	900 (530–1300)
Ferritine ng/mL, median (IQR)	1100 (609–1600)
Acute kidney disease %	75.8
ICU admission %	49.4
Invasive mechanical ventilation %	45.3
Vasopressors %	38.0
Vaccination %	54.1
In-hospital mortality %	30.2

AKD was present in 273 patients (75.8%), of whom 142 (52.0%) had only proteinuria, 11 (4.1%) had AKI without proteinuria, and 120 (43.9%) had both proteinuria and AKI. Eighty-seven patients (24.2%) did not exhibit evident kidney disease. Overall, proteinuria with or without AKI was present in 262 patients (72.8%), with 37 of them developing this condition during their hospital stay. Notably, these patients had higher D-dimer levels [1048 (900–1430) ng/mL vs. 900 (520–1250) ng/mL], worse Pa/FiO2 [80 (78–85) vs. 134 (99–180)], longer lengths of hospital stay [26 (12–45) days vs. 15 (7–25) days], and a trend toward more AKI, ICU admissions, mechanical ventilation, and mortality. On the other hand, AKI with or without proteinuria occurred in 131 patients (36.4%). The predominant pattern of AKI was severe (KDIGO 3, 47.9%), hospital-acquired (70.9%), and non-oliguric (71.4%). Kidney replacement therapy was required in 37.4% of AKI patients. Renal recovery was observed in 52.6% (full recovery in 40.2% and partial recovery in 12.4%), while non-recovery occurred in the remaining 47.4%. Table 2 presents the clinical characteristics of patients according to their AKD stage. Importantly, the mortality rate increased with the severity of AKD stage: non-kidney disease 9.5%, only proteinuria 22.8%, only AKI 56.7%, and proteinuria plus AKI 53.0% (p = 0.001). According to multivariate analysis, mortality was associated to age, obesity, AKD category, and hospital setting (Table 3). The risk of dying was higher in patients with AKI KDIGO 2 than KDIGO 3 [11.167 (2.567–48.57, p = 0.001)], [35.882 (8.852–144.70, p < 0.001)]. Vaccination status was not associated to mortality (data not shown).

**Table 2:** CLINICAL CHARACTERISTICS OF PATIENTS ACCORDING TO AKD CATEGORY

	Non AKD 87	Proteinuria 142	AKI 11	Pr + AKI 120	p
Age, years, median (IQR)	59 (42–71)	61.5 (49–76)	72 (64–79)	66 (53–74)	0.041
Hypertension %	36.8	43.7	40.0	69.0	0.000
Diabetes %	20.9	26.2	60.0	43.5	0.001
Chronic heart disease %	3.4	7.6	0	23.8	0.000
Hospital setting % Ward Emergency room ICU	87.4 2.3 10.3	73.2 10.6 16.2	72.7 18.2 9.1	64.4 16.1 19.5	0.013
Lymphocyte count mm^3^	1000 (678–1577)	835 (608–1170)	660 (580–1200)	1063 (652–2330)	0.005
Platelet count mm^3^	259 (210–311)	220 (172–292)	185 (114–287)	242 (189–308)	0.045
D-dimers ng/mL	800 (582–1000)	850 (500–1200)	695 (420–1352)	1123 (597–1477)	0.012
Ferritine ng/mL	900 (503–1200)	1141 (646–1600)	1100 (653–1680)	1500 (767–1800)	0.001
ICU admission %	27.9	42.9	72.7	70.6	0.000
Invasive mechanical ventilation %	32.4	58.7	70.0	78.8	0.000
Vasopressors %	11.1	25.0	50.0	67.6	0.000
In-hospital mortality %	5.8	24.1	36.4	55.5	0.000

**Table 3:** MULTIVARIATE STEPWISE LOGISTIC REGRESSION MODEL

	OR	95% CI	p
Age	1.038	1.017–1.049	<0.001
Obesity	2.18	1.063–4.487	0.033
AKD category Proteinuria AKI Proteinuria+AKI	4.41 6.36 20.76	1.540–12.63 1.040–38.92 7.145–60.28	0.006 0.045 <0.001
Hospital setting Ward ICU	1.972 8.32	0.674–5.774 3.792–18.56	a/s <0.001

Note – Sex, hypertension, and diabetes were not included in the model.

## Discussion

Our results endorse the use of AKD criteria as a useful tool to identify all the spectrum of functional or structural kidney involvement in a wide time frame within and beyond the 7 days of the AKI definition. We identified about half of patients who did not meet AKI criteria, but had isolated proteinuria showing worse outcomes than those with no kidney disease (mortality rate 22.8% vs 9.5%, respectively). In regard to AKI, the predominant pattern was severe, acquired during hospital stay, non-oliguric, and frequently requiring KRT. As expected, this subset of patients had the highest mortality rate.

Typically, studies addressing renal involvement in COVID-19 report data on AKI and/or proteinuria/hematuria as separate events. However, we believe that a more integrative approach is essential to identify subgroups from the perspective of AKD, as demonstrated in our study. This approach enables us to better determine the extent of renal involvement, implement preventive and therapeutic strategies, and establish a prognostic framework. As observed, the frequency of adverse events increased in the following order: non-kidney disease, isolated proteinuria, isolated AKI, and both proteinuria and AKI. Comorbidities, the severity of the condition at admission, the need for ICU care and organ support, and laboratory abnormalities also followed a similar pattern as expected.

Our patient series exhibited the typical characteristics of individuals with COVID-19 in terms of age, gender, and comorbidities. Additionally, COVID-19 disease severity was high, with approximately half of the patients requiring ICU admission, mechanical ventilation, experiencing serious complications, and having a high in-hospital mortality rate. Overall, AKD was a prevalent condition, affecting nearly 76% of patients. The most common disturbance was proteinuria, with or without AKI (72.8%), followed by AKI with or without proteinuria (36.4%). As mentioned earlier, we classified patients into four groups: non-kidney disease, only proteinuria, only AKI, and proteinuria with AKI. We included in AKD without AKI those patients who had only proteinuria. This subgroup is noteworthy because, as demonstrated by James et al.^7^ and Sawhney et al.^8^, among others, AKD without AKI is a frequent and serious occurrence. James et al. reported that the incidence of this subgroup was three times the incidence of AKI, and the expected adverse outcomes were at least as severe as those of AKI. The authors concluded that AKD without AKI is common, identifies patients not recognized by AKI and CKD criteria, and is associated with an overall increased risk of long-term adverse outcomes, as found in this study. In our series, AKD without AKI, when compared to non-kidney disease, was associated with more comorbidities, worse markers of disease severity, and an increase in in-hospital mortality from 5.8% to 24.1%. This time frame is critical for preventing worsening of the condition and progression to CKD.

Furthermore, in our series, AKI with or without proteinuria occurred in a third of cases, with the majority being severe, hospital-acquired, and non-oliguric. We observed a higher frequency of KDIGO 3, followed by KDIGO 1 and KDIGO 2 stages, as is common in this setting. The main causes of AKI were multiorgan dysfunction linked to COVID-19 or sepsis (47.6% and 25.0%, respectively), followed by hypovolemia or dehydration (22.6%). This pattern reflects the multifactorial nature of renal impairment in COVID-19-related AKI due to the effects of cytokine storms, hypovolemia, mechanical ventilation, and nephrotoxins (9). Approximately one-third of patients received kidney replacement therapy (KRT), primarily intermittent hemodialysis (IHD), despite being critically ill patients for whom continuous kidney replacement therapy (CKRT) or prolonged intermittent kidney replacement therapy (PIKRT) might have been more appropriate considering limited resources during the pandemic. None of the patients were treated with extracorporeal blood purification. A similar profile was found in two Latin American studies conducted by our group, one of which focused on COVID-19-related AKI.^10,11^ Recovery of kidney function, both complete and partial, was slightly higher in our study. However, it should be noted that follow-up was only conducted until hospital discharge or death.

Finally, we explored the association between clinical conditions and vaccination status. We obtained data for slightly more than half of the population, of which 54% received at least one dose of the vaccine. The vast majority of these patients received full vaccination (82%). Vaccinated patients had fewer comorbidities, less severe disease at admission and during their hospital stay, less proteinuria, and better outcomes. Distribution of patients within AKD categories was similar (data not shown). These findings are likely related to the positive impact of the COVID-19 vaccine in reducing the severity of infection.

Our study has certain limitations, primarily stemming from the potential bias due to the observational design of the study and the limited number of patients, which affected the power of the statistical analysis. Additionally, a high number of missing cases in some variables due to work overload because of the pandemic, prevented us from conducting a thorough analysis of the dynamics of the process, such as the trajectory of proteinuria and AKI, the time from AKI to ICU admission, and mechanical ventilation.

The primary strength of our study lies in supporting the strategy of approaching kidney disorders from the perspective of AKD. AKD should be understood as a continuous and dynamic process that enables the timely identification of patients at the early stages of kidney injury, thereby reducing the risk of further progression^12,13^. As stated by Sawhney et al.,^12^ “further work has been called for to reconcile the use of these definitions for clinicians and researchers, and to provide a common understanding of disease definitions for comparisons of the burden and outcomes of AKI and AKD across time, patient subgroups, and clinical settings”.

In conclusion, our data endorse a comprehensive approach to acute kidney diseases and disorders based on the concept of AKD. This integrative approach, encompassing the structural and functional continuum of AKI, AKD, and CKD, may allow timely interventions and the implementation of preventive and therapeutic strategies.

## Data Availability

The datasets used and analyzed in the current study were deposited at the RedIRA site https://slanh.net/red-ira/ and available from the corresponding author on reasonable request.
