# Supplementary material for: Acute kidney disease in patients with COVID-19. A prospective, multicenter, multinational study in Latin America
Source: J Bras Nefrol. 2025 Aug 18;47(3):e20240154. doi: 10.1590/2175-8239-JBN-2024-0154en (PMC12448115; doi:10.1590/2175-8239-JBN-2024-0154en)
Supplement: Translation

# Translation: Doença renal aguda em pacientes com COVID-19: um estudo prospectivo, multicêntrico e multinacional na América Latina

**DOI:** 10.1590/2175-8239-JBN-2024-0154pt

**Authors:** Raúl Lombardi, Alejandro Ferreiro, Yanissa Venegas, Mariana Pereira, Cristina Carlino, Rolando Claure-Del Granado, Daniela Ponce, Daniel Molina, Agustina Zinoveev

**Affiliations:** 1 Universidad de la República, Facultad de Medicina, Centro de Nefrología, Montevideo, Uruguay.; 2 Universidad Peruana Cayetano Heredia, Hospital Nacional Arzobispo Loayza, Lima, Perú.; 3 Hospital do Servidor Público do Estado de São Paulo, São Paulo, SP, Brazil.; 4 Hospital Provincial de Rosario, Nefrología, Santa Fe, Argentina.; 5 Ministerio de Salud, Centro Único de Donación, Ablación e Implante de Órganos (CUDAIO), Santa Fe, Argentina.; 6 Caja Nacional de Salud, Hospital Obrero No 2, División de Nefrología, Cochabamba, Bolivia.; 7 Universidad Mayor de San Simon, Facultad de Medicina, Instituto de Investigaciones Biomédicas e Investigación Social (IIBISMED), Cochabamba, Bolivia.; 8 Universidade Estadual Paulista, Departamento de Nefrologia, Botucatu, SP, Brasil.; 9 Caja Petrolera de Salud, Santa Cruz, Bolivia.; 10 Centro de Asistencia del Sindicato Médico del Uruguay (CASMU), Institución de Asistencia Médica Privada de Profesionales sin fines de lucro (IAMPP), Departamento de Nefrología, Montevideo, Uruguay.

**Keywords:** Doença Renal Aguda, Injúria Renal Aguda, COVID-19, Proteinúria

## Abstract

**Introdução:**

A doença renal aguda (DRA) é definida como anormalidades renais funcionais e/ou estruturais, com implicações para saúde e duração ≤90 dias. O objetivo foi avaliar a DRA como uma abordagem mais apropriada para essas condições, utilizando uma coorte com COVID-19, onde o comprometimento renal é expresso por proteinúria e/ou perda de função.

**Métodos:**

Estudo de coorte observacional, prospectivo, longitudinal, multinacional realizado em cinco países latino-americanos. Foram incluídos pacientes adultos com diagnóstico de COVID-19. Na internação, realizou-se urinálise ou teste de urina com tira reagente. Caso a proteína não fosse detectada, uma investigação de acompanhamento era feita cinco dias depois. Os pacientes foram classificados em quatro categorias de DRA: doença não renal, somente proteinúria, somente injúria renal aguda (IRA), proteinúria e IRA.

**Resultados:**

Foram incluídos 360 pacientes. A DRA esteve presente em 273 (75,8%), dos quais 142 (52,0%) apresentaram somente proteinúria, 11 (4,1%) IRA sem proteinúria e 120 (43,9%) apresentaram proteinúria e IRA. No geral, a proteinúria, com ou sem IRA, foi observada em 262 pacientes (72,8%). A IRA, com ou sem proteinúria, ocorreu em 131 pacientes (36,4%). A IRA foi predominantemente grave, não oligúrica e adquirida no hospital. A mortalidade hospitalar aumentou com a gravidade da DRA: doença não renal 9,5%, somente proteinúria 22,8%, somente IRA 56,7%, proteinúria mais IRA 53,0% (p = 0,001).

**Conclusões:**

Nossos dados endossam uma abordagem abrangente baseada no conceito de DRA. Essa abordagem integrativa, englobando o *continuum* estrutural e funcional de IRA, DRA e DRC, permite intervenções oportunas e a implementação de estratégias preventivas e terapêuticas.

## Introdução

O conceito de doença renal aguda foi introduzido pela primeira vez pelo Grupo de Trabalho sobre IRA do KDIGO, em 2012^1^, com o objetivo de fornecer uma abordagem clínica integrada aos pacientes que apresentam anormalidades agudas na função e na estrutura renal. O Grupo de Trabalho do KDIGO define a doença renal aguda (DRA) como a ocorrência de injúria renal aguda (IRA), uma TFGe <60 mL/min/1,73 m^2^, redução da TFGe em >35%, aumento da creatinina sérica >50% ou qualquer dano renal, no período de 7 a 90 dias após um episódio de IRA. A DRA é um conceito em construção. A proposta inicial foi revisada em 2016 pelo Grupo ADQI, propondo uma definição e critérios de estadiamento e recuperação renal, além de fazer recomendações para a prática clínica e pesquisas futuras^2^. Mais recentemente, o KDIGO organizou uma conferência de consenso com o objetivo de expandir e harmonizar as definições de DRA existentes. Consequentemente, as diretrizes do KDIGO agora definem a doença renal aguda como anormalidades funcionais e/ou estruturais dos rins que afetam a saúde e duram ≤90 dias, sendo a IRA um subconjunto da DRA. A doença renal é ainda classificada de acordo com sua causa, a gravidade das anormalidades estruturais e funcionais e a duração dessas anormalidades^3^. Essa perspectiva sobre os distúrbios renais agudos, tanto funcionais quanto estruturais, proporciona uma compreensão mais ampla dessas condições complexas. Essa abordagem inovadora busca preencher a lacuna entre IRA e DRC, identificando assim os pacientes que não atendem aos critérios de IRA e DRC, mas que compartilham o espectro de distúrbios basais. Com base nesses critérios, é possível identificar subgrupos de pacientes: DRA sem IRA, DRA com IRA e aqueles com doença renal não conhecida (NKD, por sua sigla em inglês).

O envolvimento renal na COVID-19 tem sido amplamente documentado e está frequentemente associado a desfechos adversos^4^. A proteinúria foi relatada em 28–84% dos casos^5^, enquanto a IRA afeta aproximadamente 17% dos pacientes, com uma incidência que varia consideravelmente de 0,5% a 80%, conforme indicado por uma revisão sistemática e meta-análise de 20 coortes^6^. Esse perfil clínico torna essa uma população adequada para o objetivo do estudo. Até onde temos conhecimento, não há estudos que avaliem a precisão dos critérios de DRA nesse cenário. Portanto, o objetivo de nosso estudo foi avaliar os critérios de DRA como uma abordagem mais abrangente para pacientes com envolvimento renal agudo/subagudo em pacientes com COVID-19.

## Métodos

### Protocolo do Estudo

Este é um estudo de coorte observacional, prospectivo, longitudinal e multinacional realizado de 8 de março de 2021 a 30 de abril de 2022 em cinco países da América Latina (Argentina, Bolívia, Brasil, Peru e Uruguai). Foram incluídos todos os pacientes que preencheram os critérios de inclusão do estudo. Os participantes inseriram os dados em uma plataforma online dedicada, desenvolvida para esse estudo, garantindo a confidencialidade das informações por meio da desidentificação dos dados. Uma vez inscritos na plataforma, cada paciente recebeu aleatoriamente um número de identificação exclusivo, utilizado para acessar suas informações específicas. Na admissão hospitalar, a presença de proteinúria foi avaliada por meio de urinálise ou testes com tira reagente. A proteinúria foi considerada positiva se a concentração de proteína na urina fosse ≥ 0,3 g/L em amostra isolada ou ≥ + na tira reagente. Nos casos em que nenhuma proteína foi detectada inicialmente, uma análise de acompanhamento foi realizada cinco dias depois. O registro de dados foi realizado para todos os pacientes, independentemente da presença ou ausência de proteinúria. Os critérios de inclusão abrangeram pacientes adultos com idade ≥18 anos, com diagnóstico confirmado de COVID-19 por RT-PCR de swab nasofaríngeo ou teste de antígeno para SARS-CoV-2 em swab nasofaríngeo, e que necessitaram de hospitalização.

Os critérios de exclusão consistiram em pacientes com doença renal crônica (DRC) estágio 5, aqueles em diálise crônica naquele momento ou indivíduos com rim transplantado funcionante.

### Definições

O diagnóstico e o estadiamento de IRA foram determinados com base nos critérios do KDIGO 2012, que definem a IRA como um aumento na creatinina sérica (CrS) ≥0,3 mg/dL em 48 horas ou ≥50% acima do valor basal nos 7 dias anteriores. A medição ambulatorial mais recente de creatinina, 365–368 dias antes da admissão, foi considerada como valor basal. A classificação de DRA foi baseada na definição e classificação do KDIGO 2020, que considera anormalidades na função e/ou estrutura renal com implicações para a saúde e duração de ≤3 meses^6^. A não recuperação foi considerada nos casos em que a CrS não diminuiu ou o paciente permaneceu em diálise. A recuperação total foi considerada quando a CrS diminuiu para o nível basal ou abaixo dele, e a recuperação parcial ocorreu quando a CrS diminuiu, mas não atingiu o valor basal. Classificamos a IRA como adquirida na comunidade (IRA-AC) caso o paciente apresentasse CrS elevada nas primeiras 24 horas de admissão e como adquirida no hospital (IRA-AH) caso a IRA se desenvolvesse durante a internação.

### Coleta de Dados

Os dados foram obtidos dos registros clínicos dos pacientes. As variáveis incluíram país e cidade de residência, dados demográficos, comorbidades, condição na admissão, processo de cuidados, fatores de risco e etiologia da IRA, complicações hospitalares, histórico de vacinação contra a COVID-19 e condição de alta.

### Análise Estatística

As variáveis quantitativas são apresentadas como mediana e intervalo interquartil (IIQ) de acordo com sua distribuição, enquanto as variáveis categóricas são expressas em números e proporções. O teste de Kolmogorov-Smirnov foi empregado para avaliar a distribuição dos dados. Para comparações bivariadas entre grupos, o teste do qui-quadrado foi aplicado a variáveis categóricas e o teste U de Mann-Whitney ou o teste de Kruskal-Wallis foi utilizado para variáveis quantitativas. Foram calculados os *odds ratios* e os intervalos de confiança (IC) de 95%. A estimativa de risco para prever a mortalidade foi realizada por meio do cálculo do *odds ratio* (OR) utilizando o modelo de regressão logística multivariada *stepwise*, com o respectivo IC de 95%. Todos os testes estatísticos foram bicaudais e a significância foi determinada por uma probabilidade da hipótese nula ≤5%. O processamento de dados e a análise estatística foram realizados com o *IBM SPSS Statistics Base* versão 22 (NY, EUA).

### Aprovação Ética e Consentimento Para Participar

O protocolo do estudo foi aprovado pelo Comitê de Ética do Hospital de Clínicas, Faculdade de Medicina, *Universidad de la República*, Uruguai; pelo Comitê de Ética do Hospital Provincial de Rosario, Sante Fé, Argentina, e pelo Comitê de Ética do Hospital Nacional Arzobispo Loayza, Lima, Peru. Os demais participantes foram autorizados a participar do estudo pelas autoridades de seus respectivos hospitais sob a aprovação dos Comitês de Ética supracitados. Dada a natureza observacional do estudo e a devida anonimização dos dados, conforme já mencionado, o consentimento informado não foi considerado obrigatório pelos Comitês de Ética de referência, como consta no Protocolo de Estudo aprovado.

## Resultados

Trezentos e sessenta pacientes de cinco países participaram do estudo, com a seguinte distribuição: Peru (45%), Brasil (22,5%), Bolívia (14,7%), Argentina (11,1%) e Uruguai (6,7%). A idade mediana (IIQ) da coorte foi de 64 (50–74) anos, com predominância de pacientes do sexo masculino (57,7%). As comorbidades mais prevalentes foram hipertensão arterial (49,7%), diabetes (31,8%) e obesidade (26,3%). Na admissão, a mediana (IIQ) do nível de creatinina sérica foi de 0,86 (0,68-1,07) mg/dL. Com relação à hospitalização, a maioria dos pacientes foi admitida na enfermaria (73,8%), enquanto 15,6% foram admitidos na unidade de terapia intensiva (UTI) e 10,6% na unidade de emergência. No entanto, as admissões em UTIs aumentaram para 50,9% durante a internação e 45,3% dos pacientes receberam ventilação mecânica invasiva. As complicações foram frequentes (48,9%), sendo a sepse a mais comum (27,6%). O tempo mediano de permanência hospitalar foi de 15 (7–28) dias e a taxa de mortalidade hospitalar por todas as causas foi de 30,2%. Dos 185 pacientes (51,4%) para os quais as informações sobre o status da vacinação estavam disponíveis, 100 (54,1%) haviam recebido pelo menos uma dose, enquanto 82 dos pacientes vacinados receberam duas ou três doses de vacina (82%). A Tabela 1 fornece um perfil detalhado dos pacientes de acordo com seu histórico de vacinação. Pacientes vacinados apresentaram menos comorbidades, menor gravidade da doença, particularmente em termos de proteinúria e hematúria, menor taxa de admissão na UTI e taxa de mortalidade mais baixa. A IRA também foi menos frequente, mas não atingiu diferença estatística.

**Tabela 1:** Características clínicas da população total

Idade, anos, mediana (IIQ)	64 (50–74)
Sexo masculino %	57,7
Hipertensão %	49,7
Diabetes %	31,8
Obesidade %	26,3
Insuficiência cardíaca crônica %	11,5
Doença renal crônica %	4,4
Malignidade %	4,4
Imunodepressão %	4,7
Ambiente hospitalar % Enfermaria Pronto-socorro UTI	73,8 10,6 15,6
Creatinina sérica mg/dL, mediana (IIQ)	0,86 (0,68–1,07)
Contagem de linfócitos mm^3^, mediana (IIQ)	951 (622–1415)
Contagem de plaquetas mm^3^, mediana (IIQ)	235 (181–301)
D-dímero ng/mL, mediana (IIQ)	900 (530–1300)
Ferritina ng/mL, mediana (IIQ)	1100 (609–1600)
Doença renal aguda %	75,8
Internação em UTI %	49,4
Ventilação mecânica invasiva %	45,3
Vasopressores %	38,0
Vacinação %	54,1
Mortalidade hospitalar %	30,2

A DRA esteve presente em 273 pacientes (75,8%), dos quais 142 (52,0%) apresentaram apenas proteinúria, 11 (4,1%) apresentaram IRA sem proteinúria e 120 (43,9%) tiveram proteinúria e IRA. Oitenta e sete pacientes (24,2%) não exibiram doença renal evidente. No geral, a proteinúria com ou sem IRA foi observada em 262 pacientes (72,8%), sendo que 37 deles desenvolveram essa condição durante a permanência hospitalar. Notavelmente, esses pacientes apresentaram níveis mais elevados de D-dímero [1048 (900–1430) ng/mL *vs.* 900 (520-1250) ng/mL], pior relação Pa/FiO2 [80 (78–85) *vs.* 134 (99–180)], maior tempo de permanência hospitalar [26 (12-45) dias *vs.* 15 (7–25) dias] e uma tendência de maior ocorrência de IRA, internações em UTI, ventilação mecânica e mortalidade. Por outro lado, a IRA com ou sem proteinúria ocorreu em 131 pacientes (36,4%). O padrão predominante de IRA foi grave (KDIGO 3; 47,9%), adquirida no hospital (70,9%) e não oligúrica (71,4%). A terapia renal substitutiva foi necessária em 37,4% dos pacientes com IRA. A recuperação renal foi observada em 52,6% (recuperação total em 40,2% e recuperação parcial em 12,4%), enquanto a não recuperação ocorreu nos 47,4% restantes. A Tabela 2 apresenta as características clínicas dos pacientes de acordo com o estágio da DRA. É importante ressaltar que a taxa de mortalidade aumentou com a gravidade do estágio da DRA: doença não renal 9,5%, somente proteinúria 22,8%, somente IRA 56,7% e proteinúria mais IRA 53,0% (p = 0,001). De acordo com a análise multivariada, a mortalidade foi associada à idade, obesidade, categoria de DRA e ambiente hospitalar (Tabela 3). O risco de óbito foi maior em pacientes com IRA KDIGO 2 do que KDIGO 3 [11,167 (2,567–48,57; p = 0,001)], [35,882 (8,852–144,70; p < 0,001)]. O status de vacinação não foi associado à mortalidade (dados não mostrados).

**Tabela 2:** Características clínicas dos pacientes de acordo com a categoria de DRA

	Não DRA 87	Proteinúria 142	IRA 11	Pr + IRA 120	p
Idade, anos, mediana (IIQ)	59 (42–71)	61,5 (49–76)	72 (64–79)	66 (53–74)	0,041
Hipertensão %	36,8	43,7	40,0	69,0	0,000
Diabetes %	20,9	26,2	60,0	43,5	0,001
Insuficiência cardíaca crônica %	3,4	7,6	0	23,8	0,000
Ambiente hospitalar % Enfermaria Pronto-socorro UTI	87,4 2,3 10,3	73,2 10,6 16,2	72,7 18,2 9,1	64,4 16,1 19,5	0,013
Contagem de linfócitos mm^3^	1000 (678–1577)	835 (608–1170)	660 (580–1200)	1063 (652–2330)	0,005
Contagem de plaquetas mm^3^	259 (210–311)	220 (172–292)	185 (114–287)	242 (189–308)	0,045
D-dímero ng/mL	800 (582–1000)	850 (500–1200)	695 (420–1352)	1123 (597–1477)	0,012
Ferritina ng/mL	900 (503–1200)	1141 (646–1600)	1100 (653–1680)	1500 (767–1800)	0,001
Internação em UTI %	27,9	42,9	72,7	70,6	0,000
Ventilação mecânica invasiva %	32,4	58,7	70,0	78,8	0,000
Vasopressores %	11,1	25,0	50,0	67,6	0,000
Mortalidade hospitalar %	5,8	24,1	36,4	55,5	0,000

**Tabela 3:** Modelo de regressão logística multivariada *stepwise*

	OR	IC 95%	p
Idade	1,038	1,017–1,049	<0,001
Obesidade	2,18	1,063–4,487	0,033
Categoria de DRA Proteinúria IRA Proteinúria+IRA	4,41 6,36 20,76	1,540–12,63 1,040–38,92 7,145–60,28	0,006 0,045 <0,001
Ambiente hospitalar Enfermaria UTI	1,972 8,32	0,674–5,774 3,792–18,56	n/s <0.001

Nota – Sexo, hipertensão e diabetes não foram incluídos no modelo.

## Discussão

Nossos resultados endossam o uso dos critérios de DRA como uma ferramenta útil para identificar todo o espectro de envolvimento renal funcional ou estrutural em um amplo intervalo de tempo, dentro e além dos 7 dias da definição de IRA. Identificamos aproximadamente metade dos pacientes que não preencheram os critérios de IRA, mas apresentaram proteinúria isolada, exibindo desfechos piores do que aqueles sem doença renal (taxa de mortalidade de 22,8% *vs.* 9,5%, respectivamente). Quanto à IRA, o padrão predominante foi grave, adquirida durante a internação, não oligúrica e, frequentemente, com necessidade de TRS. Como esperado, esse subgrupo de pacientes apresentou a maior taxa de mortalidade.

Normalmente, os estudos que abordam o envolvimento renal na COVID-19 relatam dados sobre IRA e/ou proteinúria/hematúria como eventos separados. No entanto, acreditamos que uma abordagem mais integrativa é essencial para identificar subgrupos a partir da perspectiva da DRA, conforme demonstrado em nosso estudo. Essa abordagem nos permite determinar melhor a extensão do envolvimento renal, implementar estratégias preventivas e terapêuticas e estabelecer uma estrutura prognóstica. Como observado, a frequência de eventos adversos aumentou na seguinte ordem: doença não renal, proteinúria isolada, IRA isolada e proteinúria e IRA combinadas. As comorbidades, a gravidade da condição na admissão, a necessidade de cuidados em UTI e suporte de órgãos e as anormalidades laboratoriais também seguiram um padrão semelhante ao esperado.

Nossa série de pacientes exibiu as características típicas de indivíduos com COVID-19 em termos de idade, sexo e comorbidades. Além disso, a gravidade da doença da COVID-19 foi alta, com aproximadamente metade dos pacientes necessitando de internação em UTI, ventilação mecânica, enfrentando complicações graves e apresentando uma taxa elevada de mortalidade hospitalar. De forma geral, a DRA foi uma condição prevalente, afetando cerca de 76% dos pacientes. O distúrbio mais comum foi a proteinúria, com ou sem IRA (72,8%), seguida por IRA com ou sem proteinúria (36,4%). Conforme mencionado anteriormente, classificamos os pacientes em quatro grupos: doença não renal, somente proteinúria, somente IRA e proteinúria com IRA. Incluímos no grupo DRA sem IRA os pacientes que apresentaram somente proteinúria. Esse subgrupo é digno de nota pois, como demonstrado por James et al.^7^ e Sawhney et al.^8^, entre outros, a DRA sem IRA é uma ocorrência frequente e grave. James et al. relataram que a incidência desse subgrupo foi três vezes superior à incidência de IRA, e os desfechos adversos esperados foram, no mínimo, tão graves quanto os da IRA. Os autores concluíram que a DRA sem IRA é comum, identifica pacientes não reconhecidos pelos critérios de IRA e DRC e está associada a um aumento geral no risco de desfechos adversos em longo prazo, conforme constatado neste estudo. Em nossa série, a DRA sem IRA, quando comparada à doença não renal, foi associada a mais comorbidades, piores marcadores de gravidade da doença e um aumento na mortalidade hospitalar de 5,8% para 24,1%. Esse período é fundamental para evitar a piora da condição e a progressão para DRC.

Além disso, em nossa série, a IRA com ou sem proteinúria ocorreu em um terço dos casos, sendo a maioria grave, adquirida no hospital e não oligúrica. Observamos uma maior frequência do estágio KDIGO 3, seguido pelos estágios KDIGO 1 e KDIGO 2, como é comum nesse contexto. As principais causas de IRA foram a disfunção de múltiplos órgãos associada à COVID-19 ou sepse (47,6% e 25,0%, respectivamente), seguidas por hipovolemia ou desidratação (22,6%). Esse padrão reflete a natureza multifatorial do comprometimento renal na IRA relacionada à COVID-19 devido aos efeitos das tempestades de citocinas, hipovolemia, ventilação mecânica e nefrotoxinas (9). Aproximadamente um terço dos pacientes recebeu terapia renal substitutiva (TRS), principalmente hemodiálise intermitente (HDI), apesar de serem pacientes críticos para os quais a terapia renal substitutiva contínua (TRSC) ou a terapia renal substitutiva intermitente prolongada (TRSIP) poderiam ter sido mais apropriadas, considerando os recursos limitados durante a pandemia. Nenhum dos pacientes foi tratado com purificação sanguínea extracorpórea. Um perfil semelhante foi encontrado em dois estudos latino-americanos realizados por nosso grupo, um dos quais focado na IRA relacionada à COVID-19.^10,11^ A recuperação da função renal, tanto completa quanto parcial, foi ligeiramente maior em nosso estudo. No entanto, deve-se observar que o acompanhamento foi realizado somente até a alta hospitalar ou óbito.

Por fim, exploramos a associação entre as condições clínicas e o status de vacinação. Obtivemos dados de pouco mais da metade da população, dos quais 54% receberam pelo menos uma dose da vacina. A grande maioria desses pacientes recebeu vacinação completa (82%). Os pacientes vacinados apresentaram menos comorbidades, doença menos grave na admissão e durante a internação, menos proteinúria e melhores desfechos. A distribuição dos pacientes dentro das categorias de DRA foi semelhante (dados não mostrados). Esses achados provavelmente estão relacionados ao impacto positivo da vacina contra a COVID-19 na redução da gravidade da infecção.

Nosso estudo apresenta algumas limitações, decorrentes principalmente do possível viés devido ao desenho observacional do estudo e ao número limitado de pacientes, o que afetou o poder da análise estatística. Além disso, um grande número de casos com dados ausentes em algumas variáveis, devido à sobrecarga de trabalho em decorrência da pandemia, nos impediu de realizar uma análise detalhada da dinâmica do processo, como a trajetória da proteinúria e da IRA, o tempo entre o início da IRA e a admissão na UTI e a necessidade de ventilação mecânica.

O principal ponto forte de nosso estudo está no suporte à estratégia de abordar os distúrbios renais sob a perspectiva da DRA. A DRA deve ser entendida como um processo contínuo e dinâmico que permite a identificação oportuna de pacientes nos estágios iniciais da injúria renal, reduzindo, assim, o risco de progressão adicional^12,13^. Como declarado por Sawhney et al.,^12^ “foi necessário uma investigação mais aprofundada para reconciliar o uso dessas definições para clínicos e pesquisadores e para fornecer um entendimento comum das definições das doenças para comparações da carga e dos desfechos de IRA e DRA ao longo do tempo, em subgrupos de pacientes e em cenários clínicos”.

Em conclusão, nossos dados apoiam uma abordagem abrangente para doenças e distúrbios renais agudos com base no conceito de DRA. Essa abordagem integrativa, que compreende o *continuum* estrutural e funcional de IRA, DRA e DRC, pode permitir intervenções oportunas e a implementação de estratégias preventivas e terapêuticas.

## Data Availability

Os conjuntos de dados utilizados e analisados no presente estudo foram depositados no site da RedIRA (https://slanh.net/red-ira/) e estão disponíveis mediante solicitação razoável ao autor correspondente.
